# P31 Frequency, resistance pattern and antibiogram of bacterial pathogens isolated in Yastebsheron Hospital, Khartoum, Sudan

**DOI:** 10.1093/jacamr/dlad066.035

**Published:** 2023-06-14

**Authors:** Mihad Altahir, Mohammed Saeed

**Affiliations:** Faculty of Medical Laboratory, UMST University, Khartoum, Sudan; Faculty of Medical Laboratory, National Ribat University, Khartoum, Sudan

## Abstract

**Background:**

This study focused on providing a report about the current situation of AMR in Yastebsheron Hospital in Khartoum, Sudan, monitoring and identifying the resistant trends in clinical isolates, highlighting the gaps in the IPC and ASP through antibiogram analysis, and implementing its data to improve antibiogram-guided empirical antibiotic therapy selection.

**Method:**

A retrospective longitudinal study. The study retrieved data from microbiology laboratory records from 1 January 2022 to 31 December 2022, at Yastebsheron Hospital. A total of 1182 clinical specimens were processed in 2022.

**Results:**

The most frequent samples were urine, with a prevalence rate of 59.3%, of which 69.8% were female and 30.2% were male. The total Gram-positives were 52.6%, with *Staphylococcus aureus* accounted for 98%; out of the total specimens, MRSA accounted for 48.6%, and MSSA accounted for 11.8%. The total Gram-negative isolates were 48.3%, of which *Escherichia coli Enterobacter*, *Proteus mirabilis*, *Pseudomonas aeruginosa* and *Klebsiella pneumoniae* represented 25.4%, 21.8%, 9.8%, 9.4% and 3.8%, respectively. MSSA were highly susceptible to meropenem (97%) and vancomycin (95%), and luckily, MRSA isolates were susceptible to levofloxacin and colistin at 86% and to meropenem (84%), with minimal susceptibility to clindamycin (37%) and nalidixic acid (12%). Meropenem, colistin and nitrofurantoin were the most effective antibiotics against *E. coli* with more than 80% susceptibility rates, while cephalosporins and nalidixic acid showed minimal efficacy. Meropenem and levofloxacin were highly effective against *Enterobacter*, *P. mirabilis*, while the susceptibility rates for second-, third- and fourth-generation cephalosporins were less than 50% for both of them, and less than 30% for *P. aeruginosa*, *P. aeruginosa* was 100% susceptible to colistin.

**Conclusions:**

Among infections, UTIs had the highest incidence rates. The majority of the isolates were *S. aureus*, then came Enterobacteriaceae, particularly *E. coli*, and then *P. aeruginosa*. Contrary to ciprofloxacin and norfloxacin, meropenem showed high efficacy against G-positive and G-negative isolates, while levofloxacin displayed moderately high results. Except for MSSA, the effectiveness of second-, third- and fourth-generation cephalosporins against G-positive and G-negative organisms was compromised. In order to stop the deterioration of the AMR status, it is crucial to implement the ASP strategies and tools effectively, as the emergence of MDR strains is alarming. Antibiogram’s formulation is essential for directing empirical antibiotic therapy and tracking the emergence of resistance.

